# Assessment of dynamic balancing responses following perturbations during slow walking in relation to clinical outcome measures for high-functioning post-stroke subjects

**DOI:** 10.1186/s12984-020-00710-5

**Published:** 2020-07-02

**Authors:** Matjaž Zadravec, Andrej Olenšek, Marko Rudolf, Nataša Bizovičar, Nika Goljar, Zlatko Matjačić

**Affiliations:** University rehabilitation institute Republic of Slovenia, Linhartova 51, SI-1000 Ljubljana, Slovenia

**Keywords:** Balance assessment, Perturbed walking, Centre-of-mass, Centre-of-pressure, Ground-reaction-force

## Abstract

**Background:**

Generating appropriate balancing reactions in response to unexpected loss of balance during walking is important to prevent falls. The purpose of this study was to assess dynamic balancing responses following pushes to the pelvis in groups of post-stroke and healthy subjects.

**Methods:**

Forty-one post-stroke subjects and forty-three healthy subjects participated in the study. Dynamic balancing responses to perturbations triggered at heel strike of the left or right leg, directed in the forward, backward, inward and outward directions during slow treadmill walking were assessed. Responses of the healthy group provided reference values used to classify responses of the post-stroke group into two subgroups; one within the reference responses (“inside” subgroup) and the other that falls out (“outside” subgroup). A battery of selected clinical outcome measures (6-Minute Walk Test, 10-Meter Walk Test, Timed-Up-and-Go test, Four Square Step Test, Functional Gait Assessment, Functional Independence Measure and One-legged stance test) was additionally assessed in the post-stroke group.

**Results:**

The “inside” subgroup of post-stroke subjects was able to appropriately modulate centre-of-pressure and ground-reaction-force both under the impaired and non-impaired leg in response to perturbations. The “outside” subgroup of post-stroke subjects showed limited modulation of centre-of-pressure and ground-reaction-force under the impaired leg; instead stepping strategy was used in which the non-impaired leg was placed such as to make a longer step (forward perturbation), to make a shorter step (backward perturbation) or to make a cross-step (outward perturbation). Consequently, peak centre-of-mass displacements following perturbations were significantly higher in the “outside” subgroup compared to the “inside” subgroup. Responses in both subgroups following inward perturbations did not differ. Majority of clinical outcome measures moderately correlated with the peak centre-of-mass displacements for forward perturbations and exhibited weak correlations for other perturbation directions.

**Conclusions:**

Substantial number of post-stroke subjects, that were considered to be independent walkers, have reduced capabilities to execute appropriate balancing responses following perturbations commencing on the hemiparetic leg and may thus benefit from perturbation-based training. Timed-Up-and-Go and Functional Independence Measure tests may provide an indication on the abilities of each subject to counteract unexpected loss of balance. However, a reliable assessment should be done through perturbation-based measures.

## Introduction

Generating appropriate balancing reactions in response to unexpected loss of balance during walking is important to regain stability and prevent falls. Several studies have applied mechanical pushes to the pelvis of walking subjects to investigate the repertoire of dynamic balancing responses [1–4]. Foot placement adjustment of the swinging leg, termed “stepping strategy”, was identified as the most important strategy to recover balance following perturbation [5]. Studies have also shown that corrective action following a perturbation starts earlier in the form of displacement of centre-of-pressure (CoP) under the stance leg in the direction of the perturbation, termed “ankle strategy” [6]. In accordance with the inverted pendulum model CoP displacement increases the horizontal component of ground-reaction-force (GRF) thus opposing the action of the perturbation [7]. The third strategy that can be used to counteract perturbation is called “inertial strategy” and is related to rotation of limb segments that modulate the horizontal component of GRF [5, 7, 8]. The ankle strategy and inertial strategy are collectively called “in-stance strategy” as they apply corrective action during the stance phase of the supporting leg and are thus much faster than the stepping strategy that can act against perturbation only once the swinging leg is placed on the ground. The stepping strategy typically prolongs the step length/width in the direction in which the perturbation acted thus also adequately modulating GRF. Matjačić et al. [9] have shown in their recent study of healthy subjects that the stepping strategy is used with walking speeds of 0.8 m/s and above while below that speed in-stance strategies are dominant.

Hemiparesis, as a result of stroke, changes the functionality of the affected body-side, which is reflected in aggravated general mobility including balance during standing and walking [10–12]. Typical hemiparetic gait is characterised by less time spent on the paretic lower extremity during walking [12], increased lateral pelvic displacements [13], asymmetrical centre-of-mass (CoM) and CoP movement patterns. Thus, we may expect that subjects following stroke, even after the completion of rehabilitation, will to some extent display impairment in their abilities to modulate CoP and GRF under the paretic leg (affecting their ability to exercise in-stance strategies) and to appropriately modify the location of the next step with their paretic leg (affecting their ability to exercise the stepping strategy). Haarman et al. [12] investigated reactive stepping following perturbing pushes applied to the waist in a group of post-stroke subjects. However, in this study the subjects walked at different, self-preferred walking speeds while perturbation amplitudes varied greatly among subjects, thus making comparison between subjects difficult. A case-control study investigating dynamic balancing responses during slow walking and after application of moderate perturbation strength in a high-functioning chronic hemiparetic subject, who was an independent walker, showed clear asymmetry between the balancing strategies used [14]; when perturbation commenced at the beginning of the stance of the paretic leg the subject used the stepping strategy, while an in-stance strategy was used when perturbed on the non-paretic leg. Clearly, there is a lack of perturbation studies which investigate dynamic balancing responses in post-stroke subjects to provide insight into the balancing strategies used at comparable walking speeds and comparable perturbation parameters.

There are no clinical outcome measures to assess the ability of post-stroke subjects to cope with unexpected perturbations during walking. Fall risk prediction was often addressed in relation to various clinical outcome measures, which provide information about the performance of post-stroke subjects in terms of motor abilities and cognitive aspects [15–17]. The Timed-Up-and-Go test (TUG), 10-Meter Walk Test (10MWT) and Functional Independence Measure (FIM) were suggested as reliable and valid assessment tools for predicting falls in moderately-functioning post-stroke subjects [18–21]. These clinical tests along with the 6-Minute Walk Test (6MWT), Functional Ambulation Category (FAC), Four Square Step Test (FSST) and many others reliably assess performance of proactive movement, including proactive balancing abilities. It is not clear to what extent these well-established clinical outcome measures can be related to the abilities of post-stroke subjects to generate appropriate reactive dynamic balancing responses following an unexpected event that challenges their stability during walking.

Reliable assessment of the ability to appropriately react to unexpected perturbations is particularly important to patients who are about to be discharged from inpatient stroke rehabilitation and are considered to be independent walkers. Such assessment would make it possible to discriminate between subjects who are able to counteract unexpected loss of balance and those with limited balancing abilities who are in need of perturbation-based training. Mansfield et al. [22] suggested that post-stroke rehabilitation strategies for fall prevention should incorporate training of dynamic balancing responses following perturbations that challenge balance by focusing on remediating dysfunction of the more affected limb. Several studies reported that perturbation-based balance training has a positive effect on improvement of reactive dynamic balancing responses following unexpected loss of balance, which might reduce the risk of falling among older adults and clinical populations [14, 23–27].

The main purpose of this study was to assess dynamic balancing responses following pushes to the pelvis of similar strength, triggered at heel strike of the paretic and non-paretic leg, during slow treadmill walking in a group of post-stroke subjects who showed ability to walk independently and were ready for discharge from an inpatient rehabilitation program. We also assessed dynamic balancing responses with the same experimental conditions for a group of neurologically intact subjects who provided reference values used to classify the responses assessed for the group of stroke subjects into two subgroups; one that falls within the reference responses and the other that falls out. A battery of selected clinical outcome measures that are routinely used in clinical practice was also assessed for a group of stroke subjects to examine the relation with the assessed dynamic balancing responses.

## Methods

### Participants

Forty-one stroke survivors (10 females, 22 with right-sided hemiparesis, age: 53.6 ± 8.7 years, body mass: 83.5 ± 16.5 kg, height: 174.3 ± 9.4 cm) and forty-three healthy adults (12 females, age: 35.7 ± 8.4 years, body mass: 74.9 ± 9.6 kg, height: 177.4 ± 6.0 cm) without known neurological, muscular or orthopedic problems, participated in this study. Assessments of dynamic balancing responses and clinical outcome measures were performed on stroke patients at the end of their hospitalisation period at the University Rehabilitation Institute Republic of Slovenia. Individual data on stroke subjects are given in Table 1. Group average data on healthy participants are given in Table 2. The inclusion criteria for stroke subjects were first unilateral stroke, independence in ambulation (functional ambulation category FAC at least 4), ability to walk independently or under supervision but without walking aids, and ability to follow instructions. All stroke subjects practiced treadmill walking during their rehabilitation program. The study was approved by the Slovenian national ethics committee and all participants provided written informed consent.

**Table 1:** Characteristics and clinical outcome measures of stroke participants

ID	Gender (M/F)	Age (yrs.)	Height (cm)	BM (kg)	Days poststroke	Stroke type	Affected side (L/R)	F_PERT_ (N)	F_PERT_ (%BW)	FAC	6MWT (m)	10MWT (m/s)	TUG (s)	FSST (s)	FGA	mFIM	OLST NP/P (s)
1	M	48	181	87	94	ISC	L	80	9.2	5	440	1.33	8.3	12.4	20	87	45/38
2	M	39	200	82	81	ISC	L	60	7.3	5	540	1.67	9.0	11.0	20	84	45/10
3	M	52	186	96	111	ISC	L	80	8.3	5	400	1.59	10.7	13.4	–	84	45/11
4	M	52	186	136	118	ISC	R	120	8.8	5	355	1.22	9.9	11.9	–	83	13/4
5	M	53	173	70	214	HEM	R	60	8.6	5	260	1.00	12.1	13.9	15	80	45/4
6	M	62	180	74	351	ISC	R	60	8.1	5	350	1.20	10.7	12.2	–	78	45/2
7	F	61	158	70	120	ISC	R	60	8.6	5	525	1.45	6.3	10.6	–	84	2/17
8	F	53	170	105	137	HEM	R	80	7.6	5	425	1.49	6.9	8.2	25	79	13/16
9	M	61	174	70	116	ISC	R	75	10.7	5	435	1.69	6.3	10.9	25	81	9/7
10	M	64	168	81	218	ISC	R	70	8.6	5	395	1.30	7.2	11.5	19	82	14/2
11	M	52	177	82	158	HEM	R	80	9.8	5	570	2.56	5.6	9.2	26	85	45/45
12	M	41	180	102	61	HEM	L	100	9.8	5	560	1.64	4.4	8.6	–	90	45/24
13	F	64	160	70	158	ISC	R	70	10.0	5	410	1.45	7.1	9.8	21	75	8/25
14	M	31	176	83	56	ISC	L	83	10.0	4	390	1.32	7.6	9.8	25	82	45/23
15	F	50	158	69	105	ISC	L	64	9.3	5	380	1.32	7.5	11.3	20	81	12/45
16	F	55	160	55	204	HEM	R	45	8.2	5	455	1.30	7.8	9.4	–	79	18/7
17	M	72	180	78	302	ISC	L	80	10.3	4	420	1.43	8.3	8.3	14	78	5/7
18	M	44	188	92	189	ISC	R	90	9.8	5	650	2.44	4.1	5.3	30	90	45/45
19	M	56	172	90	68	HEM	L	80	8.9	4	270	1.11	9.5	13.6	–	76	45/45
20	M	52	177	85	208	ISC	R	85	10.0	5	520	1.64	6.1	–	28	86	8/36
21	M	55	174	98	136	HEM	R	95	9.7	5	575	2.00	5.5	5.5	–	82	45/45
22	M	67	181	87	135	HEM	R	85	9.8	4	375	1.18	11.0	–	9	77	45/45
23	M	58	174	77	167	ISC	R	75	9.7	5	547	1.85	6.6	11.2	–	79	18/28
24	F	54	157	64	186	ISC	R	62	9.7	5	550	1.69	5.6	7.8	–	83	45/45
25	M	54	182	92	68	ISC	R	90	9.8	5	650	2.08	5.0	6.5	24	87	45/45
26	M	52	170	129	147	ISC	L	129	10.0	5	465	1.49	10.0	12.0	23	82	30/3
27	M	47	174	94	91	HEM	L	94	10.0	4	455	1.75	7.3	7.3	19	81	45/45
28	M	55	182	82	156	ISC	R	82	10.0	5	585	2.50	5.0	9.0	29	83	45/45
29	F	53	160	72	96	ISC	L	72	10.0	4	399	1.28	9.1	10.8	19	79	19/20
30	M	62	175	85	214	ISC	R	85	10.0	4	380	1.32	8.6	12.4	28	80	45/24
31	M	58	175	95	231	HEM	L	95	10.0	4	305	1.10	10.7	16.3	15	79	25/9
32	F	53	160	57	116	HEM	L	55	9.6	4	245	0.97	12.0	11.8	12	74	29/1
33	M	55	170	73	224	ISC	L	73	10.0	4	290	1.10	9.7	12.5	16	80	45/10
34	F	56	170	68	73	ISC	L	68	10.0	4	340	1.14	10.7	17.3	17	77	40/3
35	M	55	178	80	78	ISC	L	80	10.0	4	430	1.82	8.8	7.5	23	79	45/39
36	M	53	173	85	144	HEM	L	85	10.0	5	435	1.64	6.0	8.5	25	79	8/6
37	M	35	183	80	126	HEM	R	80	10.0	5	705	2.22	4.7	5.4	30	89	45/45
38	F	65	165	59	239	HEM	L	60	10.2	4	330	1.16	9.3	11.2	18	77	45/12
39	M	37	185	91	132	ISC	L	90	9.9	5	600	1.64	5.2	7.3	30	91	45/45
40	M	64	180	80	298	ISC	R	80	10.0	4	500	1.69	6.9	10.7	24	85	45/45
41	M	49	174	97	85	ISC	R	97	10.0	5	500	1.72	7.2	15.4	19	78	45/45
Mean (STD)	31 M 10 F	53.6 (8.7)	174.3 (9.4)	83.5 (16.5)	151 (70)	27 ISC 14 HEM	19 L 22 R	79 (16)	9.5 (0.8)	4.7 (0.5)	449 (112)	1.55 (0.40)	7.8 (2.2)	10.5 (2.9)	22 (6)	82 (4)	33/25 (16/17)

*BM* body mass, *BW* body weight, *NP/P* non-paretic/paretic leg, *ISC* ischemic, *HEM* hemorrhagic, *FAC* functional ambulation category, *6MWT* six minutes walking test, *10MWT* ten meters walking test, *TUG* timed up and go test, *FSST* four square step test, *FGA* functional gait assessment, *mFIM* functional independence measure (motor score), *OLST* one-legged stance test

**Table 2:** Group average characteristics of healthy participants

Gender (M/F)	Age (yrs.)	Height (cm)	BM (kg)	F_PERT_ (%BW)
31 M 12 F	35.7 (8.4)	177.4 (6.0)	74.9 (9.6)	10 (0)

### Perturbing apparatus

The Balance Assessment Robot for Treadmill walking (BART), consisting of a wide instrumented treadmill and an actuated pelvic link with pelvis brace, was used to deliver perturbing force impulses at the level of the pelvis in the mediolateral and anteroposterior directions during treadmill walking. The pelvic link interacts with the participant’s pelvis within 5 actuated degrees of freedom (DoF) – translation in the sagittal, frontal and vertical directions, pelvic rotation and pelvic list; the remaining DoF – pelvic tilt – is passive. Haptic interaction between the actuated pelvic link and the participant’s pelvis was admittance controlled in such a way that the interaction force was as low as possible (transparent mode), allowing the participant to freely move their pelvis during walking [28, 29]. Pelvis movement, measured with the pelvic link, was used to estimate movement of centre of mass (CoM) in a similar way as in our previous studies [1, 14]; ground reaction forces (GRF) and centre of pressure (CoP) signals were obtained through four precise force transducers (K3D120, ME Systeme GmbH) placed underneath the treadmill. Left and right heel strikes were identified with a custom developed algorithm using information about instantaneous CoP signals and were used as triggers to apply perturbation force. An extended description of the BART is provided in our previous studies [1, 30].

### Reactive balance assessment protocol

**Fig. 1:**
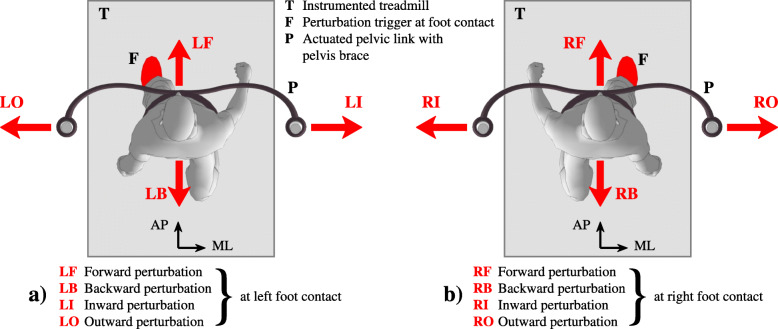
Experimental setup for assessing balance responses after perturbations applied to pelvis. Perturbations were applied in forward, backward, inward, outward directions and were triggered at either **a**) left or **b**) right heel contact

Each subject started with an introductory session lasting up to five minutes in order to familiarise themselves with the experimental set-up. All subjects were instructed to wear comfortable sport shoes. Safety was ensured with a pelvic brace, which in case of complete loss of balance would hold the subject and the treadmill immediately stopped. Subjects were secured with the pelvic brace and instructed to walk within the central area of the treadmill, which was visualised with the current pelvic position and the desired pelvis area on a screen in front of the subject. No other instructions were given to the subjects. Based on our previous studies [14] walking speed was set to 1.4 km/h (0.4 m/s) while perturbation amplitude was normalised to 10% of each participant’s body weight. These parameters were found to be acceptable to most of the stroke subjects. During the introductory session we gradually increased perturbation amplitude until 10% of body weight was reached or until the subjects became uncomfortable. The perturbation duration was set to 150 ms as in our previous studies [1, 14]. During the experiment the pelvic link of the BART was in transparent mode when no perturbing force impulses were delivered. Each participant walked for approximately 3 min at the beginning of the experiment with no perturbations delivered, which enabled us to assess their unperturbed, native gait; this initial period was followed by approximately 7 min of perturbed walking. During the perturbed walking, force impulses were triggered at either the left or right heel contact in one of four perturbation directions: forward (F), backward (B), inward (I) or outward (O). Figure 1 shows a view from the top of the experimental setup with indicated perturbation directions triggered a) on left heel contact: LF, LB, LI and LO; and b) on right heel contact: RF, RB, RI and RO. All perturbations were block-randomised. The time between two consecutive perturbations was randomly chosen from 6 to 9 s. Each perturbation was repeated 7 times, totalling as a series of 56 randomised perturbations per participant.

### Measurements and data analysis

The CoM, CoP and GRF data were first segmented into strides with the gait cycle defined as the period between two consecutive right heel strikes (for perturbations RF, RB, RI and RO) and two consecutive left heel strikes (for perturbations LF, LB, LI and LO). One gait cycle after the onset of perturbation was analysed for each perturbation direction. Similarly, one gait cycle after the left and one after the right heel strike were analysed for unperturbed walking.

Peak CoM excursions within the entire gait cycle were determined for each perturbation direction as well as for unperturbed walking. Note that these peak excursions, assessed in the direction of perturbation action, in general occur at different time instants depending on the response. The peak excursions were subtracted to yield ΔCoM variables for both planes (sagittal plane – ΔCoM_AP_; frontal plane – ΔCoM_ML_). We have also calculated time integrals of perturbed and unperturbed GRF for each perturbation direction for the “in-stance” period of the gait cycle (defined as a period between a left and right heel strike for perturbations occurring on the left leg and between a right and left heel strike for perturbations occurring on the right leg) and for the “stepping” period of the gait cycle (defined as a remaining period of the gait cycle). These integrals were normalized to body mass of each subject and were subtracted to yield force impulses for both planes (sagittal plane - ΔGRF_AP_; frontal plane – ΔGRF_ML_) which acted against perturbation in both periods of the gait cycle [9]. Since GRF determines the acceleration of CoM [7] ΔGRF can be considered as a measure of the relative share of balancing activity in both “in-stance” and “stepping” periods of balance responses [9].

CoM, CoP, GRF, ΔCoM and ΔGRF were averaged across seven repetitions for each subject for each perturbation direction and unperturbed walking. If any of the seven repetitions markedly differed it was excluded from averaging, however, in all cases at least five repetitions were averaged. A reason for excluding a particular response was typically due to the position of subject’s pelvis prior to the perturbation resulting in pelvis displacement following a perturbing push that reached the limits of BART workspace (± 25 cm), resulting in another, unwanted perturbing push.

### Assessment of clinical outcome measures

The following well-established clinical outcome measures are routinely used in clinical practice and were assessed in each stroke subject: FAC – Functional Ambulation Category, 6MWT – 6-Minute Walk Test, 10MWT – 10-Meter Walk Test (assessed at fast walking speed), TUG – Timed-Up-and-Go test, FSST – Four Square Step Test, FGA – Functional Gait Assessment, mFIM – Functional Independence Measure (motor part of the test) and One-legged stance test. Detailed descriptions of the above outcome measures can be found on the following web page [31]. The assessment of clinical outcome measures was performed within the three days following the assessment of dynamic balancing responses.

### Statistical analysis

ΔCoM responses were used to generate symmetry graphs where the data of each individual subject (stroke and healthy) were plotted in such a way that on the abscissa there was a value resulting from perturbation on the right leg while on the ordinate axis there was a value resulting from perturbation on the left leg. This was plotted for each pair of perturbation directions separately (LF and RF, LB and RB, LI and RI, LO and RO). Scores obtained in a group of healthy subjects were used to calculate covariance error ellipses with a confidence interval of 95%. The covariance error ellipse in each of the ΔCoM variables defined the classifier to create two distinct subgroups of responses in the group of stroke subjects. The “inside” subgroup was the one falling within the covariance error ellipse while the “outside” subgroup was the one falling outside of the covariance ellipse.

The normal distribution of data was tested using a Kolmogorov-Smirnov test. Two-way analysis of variance (ANOVA) was used to compare ΔCoM displacements and ΔGRF force impulses (for in-stance and stepping periods separately) between the healthy group, the “inside” and the “outside” subgroups of stroke subjects for each pair of perturbation directions (LF and RF, LB and RB, LI and RI, LO and RO). The first factor in the two-way ANOVA was a group (healthy group, “inside” subgroup and “outside” subgroup) while the second factor was perturbation onset (at non-paretic or paretic foot strike for post-stroke subjects and at left or at right foot strike for healthy subjects). The Bonferroni method was used in post-hoc comparisons. For each perturbation direction a Spearman correlation coefficient was calculated and tested for statistical significance between each assessed clinical outcome measure and respective ΔCoM assessed following perturbation commencing on the strike of hemiparetic leg. The level of significance in all statistical tests was set to 5%. Data processing and data analysis were performed in MATLAB R2018b (The MathWorks, Inc.).

## Results

Perturbation amplitudes used in dynamic balance reactions assessment are given for each individual stroke subject in Table 1. The majority of post-stroke subjects were able to tolerate perturbation amplitude set to 10% of body weight without losing a balance that would require engagement of safety harness. The assessed biomechanical data was normally distributed.

### Responses to forward perturbations

**Fig. 2:**
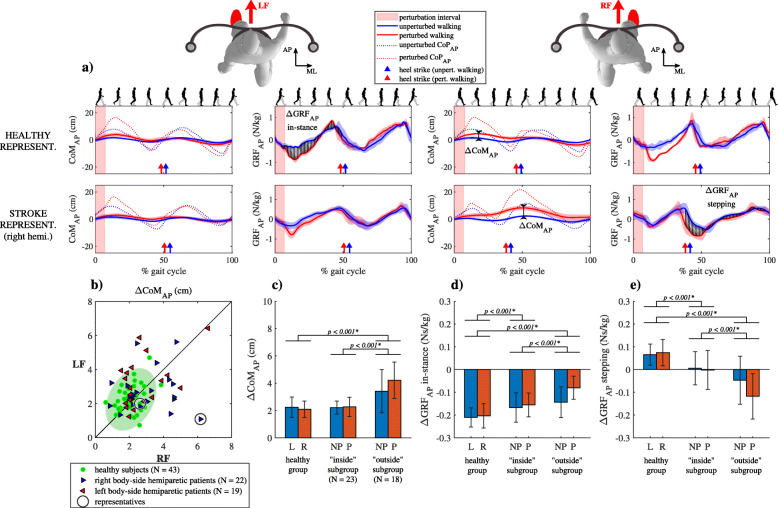
Kinematics and kinetics following forward perturbation. **a** CoM_AP_ trajectories and GRF_AP_ and CoP_AP_ signals (mean values and standard deviations) for representative healthy subject and representative right-sided hemiparetic subject over one gait cycle. **b** ΔCoM_AP_ shown for all subjects along with covariance error ellipse. **c** Group mean values and standard deviations for the ΔCoM_AP_ averaged for perturbations occurring at heel contact of left (L) or right (R) leg for group of healthy subjects and for perturbations occurring at heel contact of non-paretic (NP) or paretic (P) leg both subgroups of stroke subjects. **d** Group mean values and standard deviations for the ΔGRF_AP_ averaged for perturbations occurring at heel contact of left (L) or right (R) leg for group of healthy subjects and for perturbations occurring at heel contact of non-paretic (NP) or paretic (P) leg both subgroups of stroke subjects – “in-stance” period of response. **e** Group mean values and standard deviations for the ΔGRF_AP_ averaged for perturbations occurring at heel contact of left (L) or right (R) leg for group of healthy subjects and for perturbations occurring at heel contact of non-paretic (NP) or paretic (P) leg both subgroups of stroke subjects - “stepping” period of response. Asterisk (*) indicates a significant difference between groups in Bonferroni post-hoc paired comparisons

Figure 2a shows CoM_AP_, CoP_AP_ and GRF_AP_ trajectories for unperturbed walking and for walking after being perturbed in the forward direction (LF and RF) for a representative healthy subject and a representative right-side hemiparetic subject. Following LF perturbation CoM_AP_ trajectories were similar in both subjects; movement of CoM_AP_, which was accelerated forward by the perturbation, was decelerated backward and brought back to unperturbed values in the “in-stance” period of the gait cycle. GRF_AP_ and CoM_AP_ following LF perturbation were similar for both subjects showing forward displacement of CoP_AP_ and accompanying braking action of GRF_AP_ in the “in-stance” period of the gait cycle. Similar movement of CoM_AP_, CoP_AP_ and GRF_AP_ can be seen after RF perturbation for the healthy subject. CoM_AP_ following RF perturbation in the stroke subject showed increased forward deviation due to the action of perturbation until the next heel strike when it was finally decelerated backward and brought back to unperturbed values in the “stepping” period of the gait cycle. GRF_AP_ and CoP_AP_ signals following RF perturbation in the stroke subject were similar to those for unperturbed walking in the “in-stance” period. Increased forward displacement of CoP_AP_ and related increased braking action of GRF_AP_ can be seen in the “stepping” period.

Figure 2b shows ΔCoM_AP_ following LF and RF perturbations for all subjects where 44% of stroke subjects were in the “outside” subgroup. Majority of the subjects in the “outside” group deviated considerably from the symmetry line indicating left or right asymmetry in their responses. ΔCoM_AP_ was significantly affected by the group (F (2,162) = 47.452, *p* < 0.001) and by interaction of group and perturbation onset (F (2,162) = 3.703, *p* = 0.027) as shown in Fig. 2c. Post-hoc analysis showed greater ΔCoM_AP_ for the “outside” subgroup compared to “inside” subgroup and the healthy group.

Figure 2d and e show mean values and standard deviations for the ΔGRF_AP_ in the “in-stance” and “stepping” periods in a group of healthy subjects and for both subgroups of stroke subjects. ΔGRF_AP_ was significantly affected by the group (“in-stance” F (2,162) = 41.041, *p* < 0.001; “stepping” F (2, 162) = 56.369, p < 0.001), by perturbation onset (“in-stance” F (1,162) = 9.676, *p* = 0.002) and by interaction of both factors (“in-stance” F (2,162) = 3.711, *p* = 0.027; “stepping” F (2,162) = 3.732, *p* = 0.026). Post-hoc analysis for the “in-stance” and “stepping” periods showed significant differences in all between-group comparisons.

### Responses to backward perturbations

**Fig. 3:**
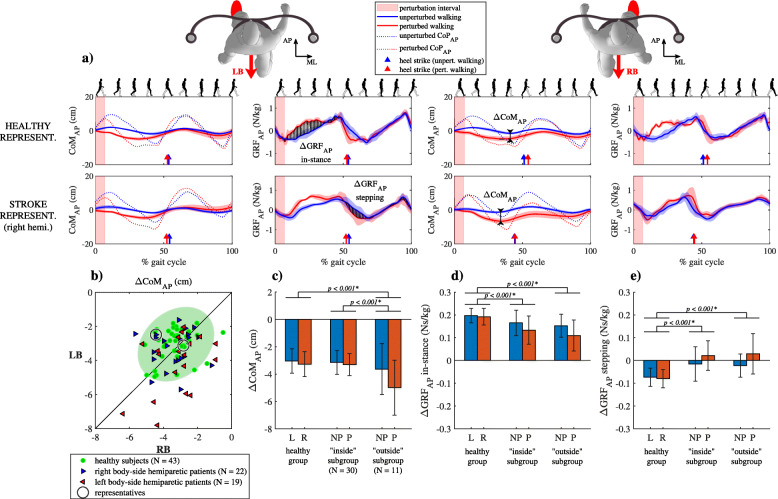
Kinematics and kinetics following backward perturbation. **a** CoM_AP_ trajectories and GRF_AP_ and CoP_AP_ signals (mean values and standard deviations) for representative healthy subject and representative right-sided hemiparetic subject over one gait cycle. **b** ΔCoM_AP_ shown for all subjects along with covariance error ellipse. **c** Group mean values and standard deviations for the ΔCoM_AP_ averaged for perturbations occurring at heel contact of left (L) or right (R) leg for group of healthy subjects and for perturbations occurring at heel contact of non-paretic (NP) or paretic (P) leg both subgroups of stroke subjects. **d** Group mean values and standard deviations for the ΔGRF_AP_ averaged for perturbations occurring at heel contact of left (L) or right (R) leg for group of healthy subjects and for perturbations occurring at heel contact of non-paretic (NP) or paretic (P) leg both subgroups of stroke subjects – “in-stance” period of response. **e** Group mean values and standard deviations for the ΔGRF_AP_ averaged for perturbations occurring at heel contact of left (L) or right (R) leg for group of healthy subjects and for perturbations occurring at heel contact of non-paretic (NP) or paretic (P) leg both subgroups of stroke subjects - “stepping” period of response. Asterisk (*) indicates a significant difference between groups in Bonferroni post-hoc paired comparisons

Figure 3a shows CoM_AP_, CoP_AP_ and GRF_AP_ trajectories for unperturbed walking and for walking after perturbation in the backward direction (LB and RB) for a representative healthy subject and a representative right-side hemiparetic subject. Following LB perturbation CoM_AP_ trajectories were similar in both subjects; movement of CoM_AP_, which was decelerated backward by the perturbation, is accelerated forward and brought back to unperturbed values in the “in-stance” period of the gait cycle. GRF_AP_ and CoM_AP_ following LB perturbation were similar for both subjects showing backward displacement of CoP_AP_ soon after the perturbation ended and accompanying accelerating action of GRF_AP_ in the “in-stance” period of the gait cycle. Similar movement of CoM_AP_, CoP_AP_ and GRF_AP_ can also be seen after RB perturbation for the healthy subject. CoM_AP_ following RB perturbation in the stroke subject showed increased backward deviation due to the action of perturbation until the next heel strike when it was accelerated forward and brought back to unperturbed values in the “stepping” period of the gait cycle. GRF_AP_ and CoP_AP_ signals following RB perturbation in the stroke subject were also similar to those after LB perturbation in the “in-stance” period, however, backward displacement of CoP_AP_ and accelerating action of GRF_AP_ were less pronounced. In the “stepping” period of the response following RB perturbation, a pronounced decrease in CoP_AP_ can be observed immediately after the heel strike of the non-paretic leg, which was related to a shortened step, with an accompanying increase of the accelerating action of GRF_AP_.

Figure 3b shows ΔCoM_AP_ following LB and RB perturbations for all subjects where 27% of stroke subjects were in the “outside” subgroup. Majority of the subjects in the “outside” group deviated considerably from the symmetry line indicating left or right asymmetry in their responses ΔCoM_AP_ was significantly affected by the group (F (2,162) = 10.735, *p* < 0.001) and by perturbation onset (F (1,162) = 9.026, *p* = 0.003) as shown in Fig. 3c. Post-hoc analysis showed greater ΔCoM_AP_ for the “outside” compared to “inside” subgroup and the healthy group.

Figure 3d and e show mean values and standard deviations for the ΔGRF_AP_ in the “in-stance” and “stepping” periods in a group of healthy subjects and for both subgroups of stroke subjects. ΔGRF_AP_ was significantly affected by the group (“in-stance” F (2,162) = 24.344, p < 0.001; “stepping” F (2, 162) = 41.105, p < 0.001), by perturbation onset (“in-stance” F (1,162) = 9.506, *p* = 0.002; “stepping” F (1, 162) = 7.055, *p* = 0.009) and by interaction of both factors (“stepping” F (2,162) = 3.697, *p* = 0.027). Post-hoc analysis for both periods has shown significant differences between the “outside” subgroup and the healthy group and between the “inside” subgroup and the healthy group.

### Responses to inward perturbations

#### Kinematics

**Fig. 4:**
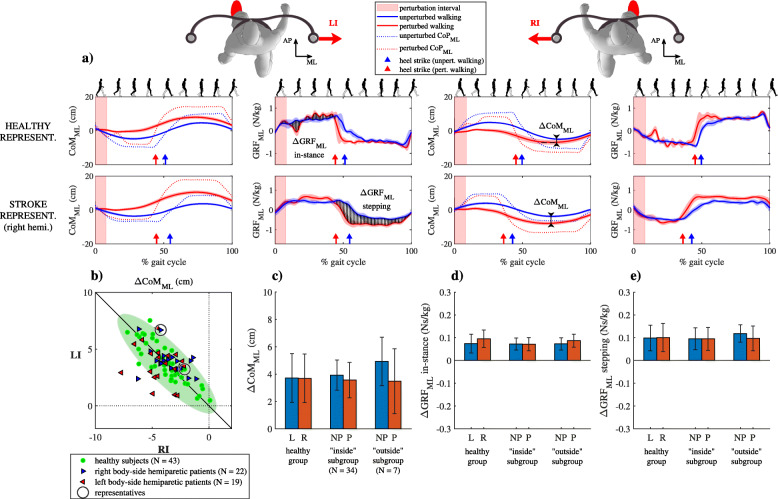
Kinematics and kinetics following inward perturbation. **a** CoM_ML_ trajectories and GRF_ML_ and CoP_ML_ signals (mean values and standard deviations) for representative healthy subject and representative right-sided hemiparetic subject over one gait cycle. **b** ΔCoM_ML_ shown for all subjects along with covariance error ellipse. **c** Group mean values and standard deviations for the ΔCoM_ML_ averaged for perturbations occurring at heel contact of left (L) or right (R) leg for group of healthy subjects and for perturbations occurring at heel contact of non-paretic (NP) or paretic (P) leg both subgroups of stroke subjects; note that a sign for red bars has been changed to facilitate visual comparison **d** Group mean values and standard deviations for the ΔGRF_ML_ averaged for perturbations occurring at heel contact of left (L) or right (R) leg for group of healthy subjects and for perturbations occurring at heel contact of non-paretic (NP) or paretic (P) leg both subgroups of stroke subjects – “in-stance” period of response; note that a sign for blue bars has been changed to facilitate visual comparison. **e** Group mean values and standard deviations for the ΔGRF_ML_ averaged for perturbations occurring at heel contact of left (L) or right (R) leg for group of healthy subjects and for perturbations occurring at heel contact of non-paretic (NP) or paretic (P) leg both subgroups of stroke subjects - “stepping” period of response; note that a sign for blue bars has been changed to facilitate visual comparison

Figure 4a shows CoM_ML_, GRF_ML_ and CoP_ML_ trajectories for unperturbed walking and for walking after perturbation in the inward direction (LI and RI) for a representative healthy subject and a representative right-side hemiparetic subject. Following LI and RI perturbations CoM_ML_ trajectories were similar in both subjects; movement of CoM_ML_, which was accelerated medially by the perturbation continued movement in the medial direction throughout the entire “in-stance” period of the gait cycle which was shortened in relation with unperturbed walking. Movement of CoM_ML_ in the medial direction was decelerated and brought back to unperturbed values in the “stepping” period. GRF_ML_ and CoP_ML_ following LI and RI perturbations were similar for both subjects showing medial displacement of CoP_ML_ in the “in-stance” period of the gait cycle. In the “stepping” period of the response following LI and RI perturbations, a pronounced increase in CoP_ML_ can be observed in the lateral direction as compared to unperturbed walking immediately after the heel strike of the non-paretic leg, which was related to a wider step with accompanying increase in accelerating action of GRF_ML_ in the medial direction.

Figure 4b shows ΔCoM_AP_ following LI and RI perturbations for all subjects where the majority of stroke subjects were in the “inside” subgroup. Figure 4c shows mean values and standard deviations for the ΔCoM_ML_ in a group of healthy subjects and for both subgroups of stroke subjects. No statistically significant interactions were found.

Figure 4d and e show mean values and standard deviations for the ΔGRF_ML_ in the “in-stance” and “stepping” periods in a group of healthy subjects and for both subgroups of stroke subjects. No statistically significant interactions were found.

### Responses to outward perturbations

**Fig. 5:**
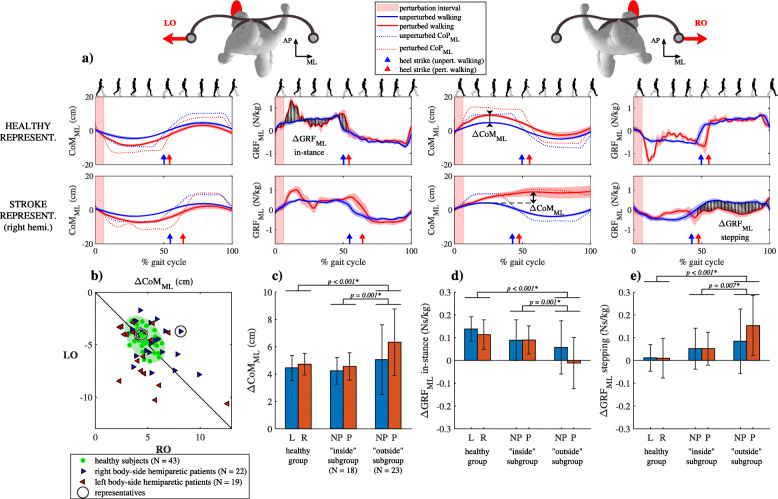
Kinematics and kinetics following outward perturbation. **a** CoM_ML_ trajectories and GRF_ML_ and CoP_ML_ signals (mean values and standard deviations) for representative healthy subject and representative right-sided hemiparetic subject over one gait cycle. **b** ΔCoM_ML_ shown for all subjects along with covariance error ellipse. **c** Group mean values and standard deviations for the ΔCoM_ML_ averaged for perturbations occurring at heel contact of left (L) or right (R) leg for group of healthy subjects and for perturbations occurring at heel contact of non-paretic (NP) or paretic (P) leg both subgroups of stroke subjects; note that a sign for blue bars has been changed to facilitate visual comparison **d** Group mean values and standard deviations for the ΔGRF_ML_ averaged for perturbations occurring at heel contact of left (L) or right (R) leg for group of healthy subjects and for perturbations occurring at heel contact of non-paretic (NP) or paretic (P) leg both subgroups of stroke subjects – “in-stance” period of response; note that a sign for red bars has been changed to facilitate visual comparison. **e** Group mean values and standard deviations for the ΔGRF_ML_ averaged for perturbations occurring at heel contact of left (L) or right (R) leg for group of healthy subjects and for perturbations occurring at heel contact of non-paretic (NP) or paretic (P) leg both subgroups of stroke subjects; note that a sign for red bars has been changed to facilitate visual comparison. Asterisk (*) indicates a significant difference between groups in Bonferroni post-hoc paired comparisons

Figure 5a shows CoM_ML_, GRF_ML_ and CoP_ML_ trajectories for unperturbed walking and for walking after perturbation in the outward direction (LO and RO) for a representative healthy subject and a representative right-side hemiparetic subject. Following LO perturbation CoM_ML_ trajectories were similar for both subjects; movement of CoM_ML_, which was accelerated laterally by the perturbation was decelerated and brought back close to unperturbed values in the “in-stance” period of the gait cycle. GRF_ML_ and CoM_ML_ signals following LO perturbations were similar for both subjects; CoP_ML_ was displaced laterally throughout the “in-stance” period of the gait cycle, which was substantially lengthened in relation with unperturbed walking; GRF_ML_ showed a substantial impulse increase immediately after the perturbation ended. GRF_ML_ and CoM_ML_ following LO perturbation in the “stepping” period were similar to unperturbed walking for both subjects. Similar movement of CoM_ML_, GRF_ML_ and CoP_ML_ can also be seen after RO perturbation for the healthy subject. CoM_ML_ following RO perturbation in the stroke subject showed increased lateral deviation due to the action of the perturbation until the next heel strike when it was decelerated in the “stepping” period of the gait cycle. GRF_ML_ and CoP_ML_ signals following RO perturbation in the stroke subject were similar to those in unperturbed walking in the “in-stance” period. In the “stepping” period of the response increase in medial displacement of CoP_ML_ can be observed, which was related to a cross-step, with accompanying increase in accelerating action of GRF_ML_ in the medial direction.

Figure 5b shows ΔCoM_ML_ following LI and RI perturbations for all subjects where 56% of stroke subjects were in the “outside” subgroup. Majority of the subjects in the “outside” group deviated considerably from the symmetry line indicating left or right asymmetry in their responses. Additionally, some of them showed symmetrical responses indicating inferior responses regardless of perturbation timing. ΔCoM_ML_ was significantly affected by the group (F (2,162) = 10.103, *p* < 0.001) and by perturbation onset (F (1,162) = 6.277, *p* = 0.013) as shown in Fig. 5c. Post-hoc analysis showed greater ΔCoM_ML_ for the “outside” subgroup compared to “inside” subgroup and the healthy group.

Figure 5d and e show mean values and standard deviations for ΔGRF_ML_ in the “in-stance” and “stepping” periods in a group of healthy subjects and for both subgroups of stroke subjects. ΔGRF_ML_ was significantly affected by the group (“in-stance” F (2,162) = 23.695, p < 0.001; “stepping” F (2, 162) = 18.699, p < 0.001) and by perturbation onset (“in-stance” F (1,162) = 5.190, *p* = 0.024). Post-hoc analysis for the “in-stance” and “stepping” periods has shown significant differences between “outside” and “inside” subgroups and between the “outside” subgroup and the healthy group.

### Clinical outcome measures

Scores on a battery of clinical outcome measures assessed in the group of stroke subjects are given in Table 1. All subjects were assessed within a window of two days after the dynamic balance responses assessment. FSST scores of two subjects and FGA scores of ten patients were not assessed as they started to feel slight dizziness in the course of assessment.

**Fig. 6:**
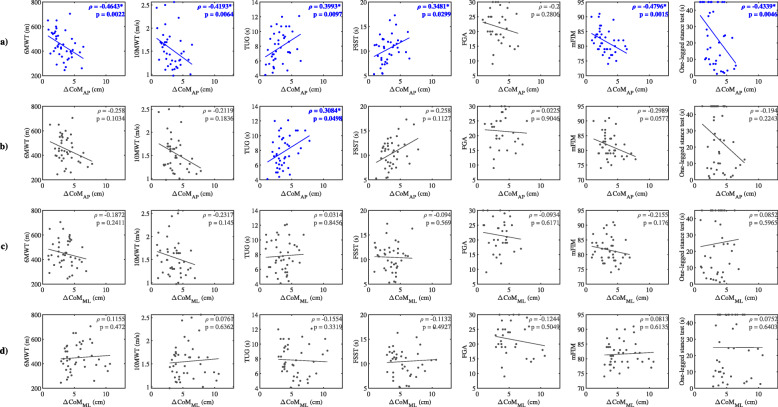
Scatter plots of 6MWT, 10MWT, TUG, FSST, FGA, mFIM and One-legged Stance Test and the corresponding ΔCoM_AP_ for **a**.) forward and **b**.) backward perturbations and ΔCoM_ML_**c**.) for inward and **d**.) outward perturbations assessed in the group of post-stroke subjects together with Spearman correlation coefficients and the corresponding *p* values. Asterisk (*) indicates statistical significance

Clinical outcome scores and the corresponding ΔCoM_AP_ (for forward and backward perturbations) and ΔCoM_ML_ (for inward and outward perturbations) assessed in the group of post-stroke subjects are displayed as scatter plots together with Spearman correlation coefficients (Fig. 6). Predominantly moderate and statistically significant relationship (Spearman correlation coefficient between 0.4–0.59) exists between the ΔCoM_AP_ assessed following the perturbations commencing on the hemiparetic leg directed forward and the majority of clinical outcome measures. Correlation of clinical outcome measures and the ΔCoM_AP_ (backward perturbations) and ΔCoM_ML_ (inward and outward perturbations) predominantly showed insignificant weak (Spearman correlation coefficient between 0.2–0.39) or very weak correlation (Spearman correlation coefficient between 0 and 0.19).

## Discussion

In this study we investigated dynamic balancing responses to perturbations applied to the pelvis in the sagittal and frontal planes during slow treadmill walking in a group of healthy subjects and a group of high-functioning stroke subjects who had completed an inpatient rehabilitation program and were independent walkers. The results showed that even though the group of stroke subjects were high-functioning independent walkers their strategies to react to unexpected loss of balance during walking differed considerably in relation to the group of healthy subjects. Clinical outcome measures routinely used in clinical practice were moderately correlated only with the balancing responses following perturbations applied in the forward direction.

### Dynamic balancing responses

The dynamic balancing responses assessed for the healthy group of subjects have shown that at the tested speed of walking and when perturbation occurred during double stance, the “in-stance” balancing strategy was used following perturbations directed forward, backward and outward. This has been reflected in modulation of CoP and GRF under the stance leg as has been previously shown in studies [4, 9]. Acting against perturbation during the stance phase is efficient as it minimises deviation of CoM and restores stability quickly [2]. The “inside” subgroup of stroke subjects was able to appropriately modulate CoP and GRF both under the impaired and non-impaired leg. The “outside” subgroup of stroke subjects did not show such a modulation under their impaired leg, thus their response mainly consisted of the “stepping” strategy where they placed their non-impaired leg so as to make a longer step (forward perturbation), a shorter step (backward perturbation) or a cross-step (outward perturbation). Since the corrective action started with a considerable delay, a higher CoM displacement resulted, which is in agreement with findings from other studies [2, 5, 9, 14]. When a perturbation was directed inward healthy subjects reacted with a stepping response, making the next step wider [4, 5, 9]. Our results show that both subgroups of stroke subjects have shown the ability to make a wider step with both legs, which is consistent with the findings of Haarman et al. [12].

It appears that the most demanding perturbations to cope with for some high-functioning stroke subjects are outward perturbations according to the highest percentage of subjects that constituted “outside” subgroup, followed by forward and backward perturbations. These three perturbation directions in contrast to inward direction demand at the tested walking speed appropriate modulation of CoP and GRF under the leg in stance, which seems to be more impaired in the “outside” subgroup of stroke subjects.

### Clinical outcome measures

The results of our study show that in a high-functioning group of stroke subjects that are independent walkers, majority of the assessed clinical outcome measures were moderately related to the abilities of responding to perturbations commencing on the paretic leg and directed forward. As the other perturbation directions are concerned weak and statistically insignificant relations were found indicating that these outcome measures do not provide information on abilities to appropriately react to unexpected loss of balance during slow walking in the backward, inward and outward directions. Some prospective studies have suggested that the TUG clinical outcome measure may be a good predictor of a potential fall in the stroke population [16, 32]. The results of our study have shown that TUG was to some extent related to the abilities of the tested group of post-stroke subjects to react to forward and backward perturbations where the obtained Spearman correlation coefficients were statistically significant. However, our results show that TUG is not related to the abilities to react to unexpected loss of balance following inward and outward perturbation.

### Methodological considerations

In this study we have selected a relatively low walking speed for assessment of dynamic balancing responses. It has been reported that the majority of post-stroke subjects walk within a walking speed range of 0.4 m/s to 0.8 m/s [33]. We have thus selected a treadmill speed of 0.4 m/s since it was experimentally determined that this was the speed at which most high-functioning stroke subjects who were about to be discharged from the inpatient rehabilitation program were comfortable. Another aspect related to the choice of suitable walking speed is that starting and stopping of walking as well as changing directions are all manoeuvres that are typically performed at reduced walking speed. Weerdesteyn et al. [34] have reported that the majority of falls in community-dwelling post-stroke subjects occurred during indoor walking activities where the speed of walking is typically low and incorporates frequent acceleration, deceleration and change of direction. It has also been shown that chronic stroke subjects needed significantly more time to accomplish the TUG test when they were required to make a turn toward their impaired side indicating reduction in their walking speed while performing a turning maneuver [35]. Thus, it seems reasonable to have investigated dynamic balancing responses to perturbing pushes during slow walking.

Previous studies [2, 9] have shown that a perturbation amplitude equalling 10% of body weight is strong enough to elicit substantial imbalance during walking without exciting leg pivoting or arm and trunk movement. The choice of eliciting perturbations at the beginning of a stance phase was motivated by the observation that this elicits use of the “in-stance” strategy to the largest extent [9]. While the results of this study would be different if another set of experimental parameters was used it is our opinion that the set used appropriately challenged the group of stroke subjects and enabled equal testing conditions.

The BART was controlled such that the interaction forces between the walking subject and the pelvis link were as low as possible. We have assessed interaction forces in a previous study and found that the influence of these forces on CoP and GRF in the sagittal and frontal planes as well as on the EMGs of major lower limb muscles during unperturbed walking had negligible effects in walking speeds ranging from 0.4–0.8 m/s [28]. In another study we have demonstrated that interaction between the balance assessment robot and the pelvis of a walking subject is purely passive, meaning that there is no exchange of energy between a walking subject and the BART except for the period when a perturbing push is delivered [1]. Thus, we may consider that the method used to deliver perturbations to the pelvis of walking subjects had negligible effects on the presented results.

### Limitations of the study

Only high-functioning post-stroke subjects who were independent walkers were included in this study as it would be very difficult to impose the perturbation protocol used on subjects with more pronounced walking impairments. Also, not all subjects were able to tolerate perturbation magnitudes of 10% of their body weight, which may have some influence on the results. Thus, the findings of this study cannot be generalised.

The mean age in the group of healthy subjects was considerably lower than that of the group of stroke subjects, since the healthy volunteers were mainly our colleagues from the rehabilitation institute. This may have affected the characteristics of the covariance error ellipses that were used to form the “inside” and “outside” subgroups of stroke subjects. Age-related balance changes and stepping reactions during walking, especially in the frontal plane, have been reported [10, 36]. Thus, older healthy subjects could exhibit different amplitudes of dynamic balancing responses. However, given the difference in performance of both subgroups of stroke subjects where the “outside” group showed clear difference in the balancing strategy used (“stepping strategy” versus “in-stance strategy”) it is our opinion that the results would not change substantially if the covariance error ellipses were somewhat different.

The battery of clinical outcome measures used in this study consisted only of tests that can be applied in a reliable and timely manner in everyday clinical practice. There exist more specialised tests to assess balance performance in stroke subjects such as the Berg Balance Scale (BBS) or the Balance Evaluation Systems Test (BEST) [37]. However, these tests are lengthy and require considerable time to administer. Additionally, these tests display a ceiling effect when applied to high-functioning post-stroke subjects. In the study of Haarman et al. [12] who investigated stepping responses in a group of stroke subjects comparable to the subjects in our study, the group BBS score was 54 of a maximum 56 points. Therefore, the BBS and BEST tests were not considered in our study.

## Conclusion

Approximately half of the high-functioning post-stroke subjects, that are considered to be independent walkers and were included in our study, have substantially reduced capabilities to execute “in-stance” balancing responses following perturbations commencing on the hemiparetic leg and directed forward, backward and outward. Even though they could counteract forward, backward and outward perturbations by utilizing stepping responses this came at the expense of significantly larger CoM deviations, making them potentially more fall-prone.

We have shown that the majority of the tested clinical outcome measures associated with balancing abilities were moderately related to dynamic balancing responses following forward-directed perturbing pushes to the pelvis commencing on the hemiparetic leg. We have further shown weak correlation between clinical outcome measures and dynamic balancing responses following perturbations in the other three directions. Clinical outcome measures, such as 6MWT, 10 MWT, mFIM, TUG and One-legged stance test could be used in clinical practice in post-stroke subjects who are independent walkers, to get an indication of the abilities of each particular subject to counteract an unexpected loss of balance. This may be relevant for the identification of those subjects who could benefit from perturbation-based training. However, a reliable assessment should be done through perturbation-based measures.

## Data Availability

The data used in this study may be available by the corresponding author upon a reasonable request to any qualified researcher.

## Abbreviations

CoM: Centre-of-mass
CoP: Centre-of-pressure
GRF: Ground-reaction-force
ΔCoM: Difference in peak values of CoM between perturbed and unperturbed walking
ΔCoP: Difference in peak values of CoP between perturbed and unperturbed walking
ΔGRF: Difference in GRF integrals between perturbed and unperturbed walking
AP: Antero-posterior
ML: Medio-lateral
BART: Balance assessment robot on treadmill
FAC: Functional Ambulation Category
6MWT: 6-Minute Walk Test
10MWT: 10-Meter Walk Test
TUG: Timed-Up-and-Go test
FSST: Four Square Step Test
FGA: Functional Gait Assessment
mFIM: Functional Independence Measure (motor part of the test)
OLST: One-legged stance test
FW: Forward
BW: Backward
IW: Inward
OW: Outward
LF: Forward perturbation triggered at left foot strike
RF: Forward perturbation triggered at right foot strike
LB: Backward perturbation triggered at left foot strike
RB: Backward perturbation triggered at right foot strike
LI: Inward perturbation triggered at left foot strike
RI: Inward perturbation triggered at right foot strike
LO: Outward perturbation triggered at left foot strike
RO: Outward perturbation triggered at right foot strike
